# The Effect of Perioperative E-Health Interventions on the Postoperative Course: A Systematic Review of Randomised and Non-Randomised Controlled Trials

**DOI:** 10.1371/journal.pone.0158612

**Published:** 2016-07-06

**Authors:** Eva van der Meij, Johannes R. Anema, René H. J. Otten, Judith A. F. Huirne, Frederieke G. Schaafsma

**Affiliations:** 1 Department of Obstetrics and Gynaecology, VU University Medical Center, Amsterdam, The Netherlands; 2 EMGO Institute for Health and Care Research, VU University Medical Center, Amsterdam, The Netherlands; 3 Medical Library, Vrije Universiteit, Amsterdam, The Netherlands; Cardiff University, UNITED KINGDOM

## Abstract

**Background:**

E-health interventions have become increasingly popular, including in perioperative care. The objective of this study was to evaluate the effect of perioperative e-health interventions on the postoperative course.

**Methods:**

We conducted a systematic review and searched for relevant articles in the PUBMED, EMBASE, CINAHL and COCHRANE databases. Controlled trials written in English, with participants of 18 years and older who underwent any type of surgery and which evaluated any type of e-health intervention by reporting patient-related outcome measures focusing on the period after surgery, were included. Data of all included studies were extracted and study quality was assessed by using the Downs and Black scoring system.

**Findings:**

A total of 33 articles were included, reporting on 27 unique studies. Most studies were judged as having a medium risk of bias (n = 13), 11 as a low risk of bias, and three as high risk of bias studies. Most studies included patients undergoing cardiac (n = 9) or orthopedic surgery (n = 7). All studies focused on replacing (n = 11) or complementing (n = 15) perioperative usual care with some form of care via ICT; one study evaluated both type of interventions. Interventions consisted of an educational or supportive website, telemonitoring, telerehabilitation or teleconsultation. All studies measured patient-related outcomes focusing on the physical, the mental or the general component of recovery. 11 studies (40.7%) reported outcome measures related to the effectiveness of the intervention in terms of health care usage and costs. 25 studies (92.6%) reported at least an equal (n = 8) or positive (n = 17) effect of the e-health intervention compared to usual care. In two studies (7.4%) a positive effect on any outcome was found in favour of the control group.

**Conclusion:**

Based on this systematic review we conclude that in the majority of the studies e-health leads to similar or improved clinical patient-related outcomes compared to only face to face perioperative care for patients who have undergone various forms of surgery. However, due to the low or moderate quality of many studies, the results should be interpreted with caution.

## Introduction

In recent years e-health interventions have become increasingly popular in medical care [1; 2].

On the one hand this is because there is a growing demand for electronic technologies in society; the development of these technologies gives people the opportunity to get information and to self-manage all type of activities in daily living, including their health [3]. On the other hand, e-health may also prove to be of great benefit to health care. It may help to deliver more patient centered care and to involve patients more in their own treatment. Better patient engagement is a crucial factor for improving quality of care and can lead to increased patient safety. It has the potential to motivate people and to turn them into more active and effective managers of their own health [4]. For this reason, also in peri-operative care e-health interventions are broadly applied [5; 6]. They are used pre-operatively with the aim to prepare patients in the best possible manner for surgery or to speed up recovery post-operatively [7–9]. Educational or supportive websites are frequently used to suit this purpose. In addition, many e-health interventions are used intra-operatively, for example tools to assist the surgeon during surgery or simulation interventions for educating trainee surgeons [10; 11]. Finally in the post-operative course e-health devices or programs are broadly applied to assist patients in their recovery process [12; 13]. This is also delivered by educational or supportive websites, but several other types of e-health interventions have been developed. For example, telemonitoring, in which patients are monitored from a distance, or telerehabilitation in which patients are supported by e-health devices in their recovery process instead of within a rehabilitation center or physiotherapy sessions in a conventional way. Finally e-consultations rather than the standard postoperative consults are applied.

E-health interventions focusing on recovery are an important topic since literature shows that recovery after surgery takes much longer than expected [14–17]. Given the growing number of surgeries per year, it is important that we find a way to support these patients in their recovery process. There are two different reasons to use e-health in perioperative care. The first one is to optimise the recovery process by providing additional care. This is evaluated by patient-related outcome measures such as satisfaction, pain or functioning. Another reason to apply e-health interventions is to substitute the usual care by some form of e-health, with the aim of delivering more efficient care. This is evaluated by outcome measures such as costs or health care usage.

Many studies have been carried out to evaluate the potential benefit of e-health interventions on the postoperative course, focusing on a wide range of surgery types, interventions and outcome measures. However, until now, no systematic review of these e-health interventions has been carried out to report the effectiveness of these types of mediation compared to more conventional perioperative care. Therefore we conducted a systematic review with the objective to evaluate the effect of perioperative e-health interventions on the postoperative course including both randomised and non-randomised controlled trials.

## Methods

We conducted a systematic review in accordance to the Prisma guidelines [18]. No protocol was registered in advance.

### Eligibility Criteria

Studies fulfilling the following inclusion criteria were included.

#### Type of Studies

We included controlled studies, containing both randomised and non-randomised comparative studies. Studies which did not include a control group drawn from the same population were excluded. The studies must have been written in English.

#### Type of Participants

Participants of 18 years and older, undergoing any type of surgery were considered.

#### Type of Interventions

Studies were included if they evaluated any type of e-health interventions. We used the definition of e-health which was defined by Paglari et al: “eHealth is an emerging field of medical informatics, referring to the organization and delivery of health services and information using the Internet and related technologies” [19]. We defined related technologies as modern technologies such as mobile apps or tele-monitoring. Interventions consisting of audiotapes or telephone calls were not considered. We only included studies in which the intervention started before surgery or within the four weeks after surgery.

#### Type of Outcome Measures

We counted studies with all types of patient-related outcome measures, including costs, with a focus on the period after surgery. Health outcomes specific for the type of surgery, and outcome measures related to knowledge or education were not considered.

### Information Sources

A systematic literature search was performed by RO and EM in the bibliographic databases PubMed, Embase.com, the Cochrane Library (via Wiley) and CINAHL (via EBSCO) from inception until the 2^nd^ of December 2015.

### Search

Search terms expressing e-health were used in ‘AND’ combination with search terms comprising the operative period. Search terms included controlled terms (e.g. MeSH in PubMed and Emtree in Embase) as well as free text terms. We used free text terms only in The Cochrane Library. The full search strategies for all the databases can be found in S1 Text. The selected studies were checked for related citations in PubMed and cross-references.

### Study Selection

Two reviewers (EM and FS) independently screened the records that were produced in the search. First, titles were screened according to the inclusion criteria. Second, the abstracts of the remaining records were screened for inclusion. The full text of the remaining articles was reviewed by both reviewers. Hereafter a third reviewer (JA) was consulted when there was disagreement about the in- or exclusion of articles by the first two reviewers. The final decision was based on consensus between the three reviewers. When articles were identified that reported the same study, initially only the parent study was included. The articles were included as separate articles when relevant outcome measures were reported or when subgroup analyses were carried out which reported results which were in line with the aim of this review.

### Data Collection Process

One reviewer (EM) extracted the data using a data extraction form which was developed by the authors, based on the Cochrane Consumers and communication Review Group’s data extraction template [20]. A second reviewer (FS) checked the extracted data. Disagreements were discussed and when necessary a third reviewer (JA) was consulted. Authors were contacted in the case of missing data.

### Data Items

Data were extracted from each included study on: 1) specific study characteristics (authors, year of publication, geographic location, study design and number of participants) 2) characteristics of the study participants (in- and exclusion criteria, reason for surgery (benign or malign), type of surgery, age, gender) 3) type of intervention (type, moment of commencement (before surgery, during hospitalisation or during or shortly after discharge), duration of the intervention) 4) type of control group and 5) outcome (type of outcome measure, methods of assessing outcome measures, timing of assessing outcome measures, follow-up duration)

### Assessment of Risk of Bias in Included Studies

Risk of bias of the individual studies was assessed by using the Downs and Black scoring system [21]. This item scoring list was adapted slightly by the authors of this review, in a similar way to previous reviews. (S1 Form) [22; 23]. We changed the answering options of item 27 ‘Did the study have sufficient power to detect a clinically important effect where the probability value for a difference being due to chance is less than 5%?’. We defined the answering options as ‘Yes’ when a power calculation was performed and there was sufficient power, ‘No’ when a power calculation was performed, but the power was not reached or a subsample was drawn from another study and ‘UTD’ when there was no report of a power calculation. The maximum score for this adapted list was 27 points Two reviewers (EM and FS) independently judged the risk of bias of the included studies. Furthermore, the two reviewers discussed about the items which were not judged the same, until they reached consensus. We defined the following three quality score classifications; good (21–27), fair (14–20) and poor (lower than 14).

### Quantitative Analysis

Due to heterogeneity in terms of type of surgery, type of intervention, type of outcome measures and study design it was not possible to conduct a meta-analysis. Instead, we aimed to present a descriptive overview of the different studies including their characteristics and results.

## Results

### Results of the Search

**Fig 1:**
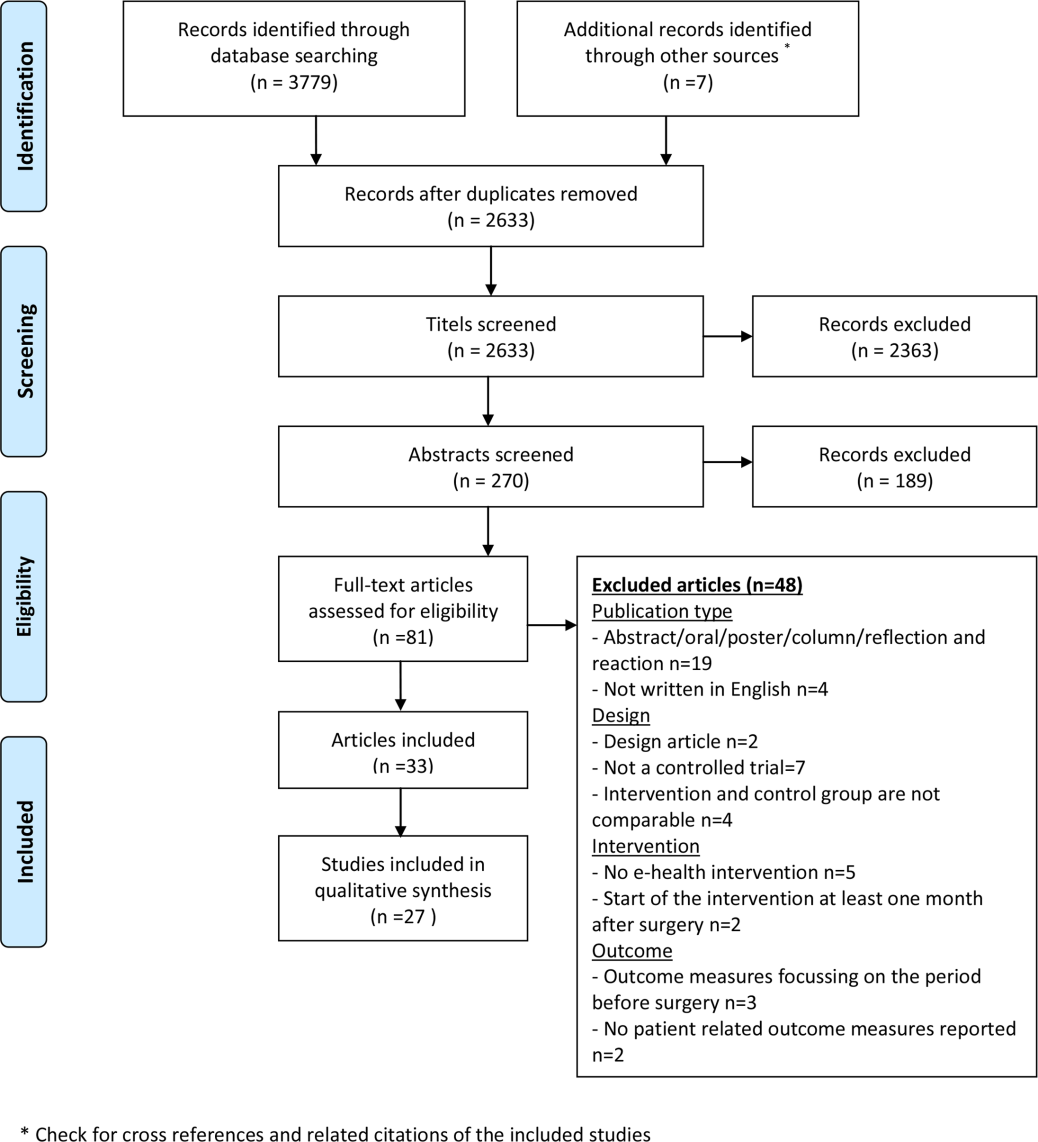
Prisma Flow diagram.

The literature search yielded 3779 records (Fig 1). Seven additional articles were identified by screening the selected studies for cross-references and related citations in Pubmed. Duplicates were removed and the titles of the remaining 2633 records were screened. After reviewing the abstracts of the remaining articles, 189 records were excluded because they did not meet the inclusion criteria. The full text of the remaining 81 articles was examined, which resulted in 33 articles fulfilling the inclusion criteria of this review, reporting on 27 unique studies (six articles reported other outcome measures or subgroup analyses of one of the included studies).

### Study Characteristics (Table 1)

#### Design of the Included Studies

Of the 27 included studies, most studies (n = 22) were randomised controlled trials; of these trials three had a non-inferiority design. The remaining five studies were prospective or retrospective controlled studies. Almost all studies had two arms, (intervention and control) except for one study with three arms [24]. Duration of follow-up varied from 24 hours [25] up to 12 months [26]. Studies were executed in 12 different countries; most of them in the USA (n = 11), followed by four in Canada. The mean number of participants per study was 130 (range 22–379) [27; 28].

**Table 1:** Study characteristics.

**Study characteristics**		**Patients**		**Intervention**			**Control**	**Outcome**			
**ID**	**Design**	**Type of surgery**	**N**	**Description of the intervention**	**Aim** ^**1**^	**Start of the intervention**	**Control group**	**Type of outcome measure**	**Follow-up until**	**Quality score** ^**2**^	**Result** ^***3***^
*Education or supportive website or device (ESW)*											
Neary et al, 2010 [25]	RCT	Minimally invasive para-thyroidectomy	64	Website with a clear stepwise description of the expected clinical course. Including the possibility to request more information or to get in contact with a member of the surgical team by e-mail	A	Before surgery	Standard website with limited information	1. Anxiety 2. Postoperative pain3. Analgesia requirements 4. Satisfaction with capacity to consent 5. Satisfaction with the intervention	24h following surgery	24	x
Vonk et al, 2014 [32]	RCT	Laparoscopic adnexal surgery or hysterectomy	215	Website with recovery instructions, tools to improve communication with care providers and to identify recovery problems	A	Before surgery	Placebo website	1. Return to work 2. Pain3. Quality of life 4. Recovery	26 weeks after surgery	25	+
Martorella et al, 2012 [37]	RCT	Cardiac surgery	60	Web-based nursing intervention (including a preoperative web-based session, 2 face-to-face postoperative sessions and it generates messages according to patients' beliefs and attitudes)	A	A few days or the day before surgery	Usual care	1. Pain intensity 2.Pain interference with daily activities 3.Patients pain barriers 4.Tendency to catastrophe pain 5. Analgesic consumption	7 days after surgery	24	+
Yin et al, 2015 [40]	RCT	Knee arthroscopy	64	Web based multimedia tutorial in addition to standard counselling. Including anatomy, pathology, and general perioperative instructions.	A	Before surgery	Usual care (standard pre-operative visit)	1. Satisfaction with overall treatment 2. Satisfaction with the intervention 3. Anxiety 4. Knowledge	First postoperative visit	22	+
Heikkinen et al, 2012 [8]	RCT	Shoulder or knee arthroscopy	147	Internet based patient education program (Containing instructions for preparing for surgery, follow-up care and financial aspects)	S	Before surgery	Face to face education with a nurse	1. Symptoms during the surgical process 2.Costs *** 3. Emotions ****	4 weeks after surgery	21	x
Miller et al, 2007 [38]	RCT	Coronary artery bypass graft surgery	49	Daily sessions with a telehealth device (focussing on assessment of symptoms, strategies to manage reported symptoms and education)	A	Discharge	Usual care	1. Physical functioning 2. Physical activities 3. Psychosocial functioning	3 months after discharge	18	x
Zimmerman et al, 2004 [39]	RCT	Coronary artery bypass graft surgery	45	Daily sessions with a telehealth device (provides health related information and recommendations related to management of recovery)	A	Discharge	Routine care	1. Symptom experience 2. Postoperative problems	6 months after discharge	16	+
Barnason et al, 2003 [34]	RCT	Coronary artery bypass graft surgery	35	Daily sessions with a telehealth device (provides strategies to manage symptoms, education and positive reinforcement)	A	Discharge	Routine care	1. Self-efficacy 2. Cardiovascular risk factor modification adherence 3.Functional status	3 months	15	+
Barnason et al, 2006 [33]	RCT	Coronary artery bypass graft surgery	50	Daily sessions with a telehealth device (provides strategies to manage symptoms, education on the recovery process and positive reinforcement)	A	Discharge	Home health care	1. Physiologic and psychosocial functioning 2. Postoperative problems 3. Health care use	3 months	15	+
Barnason et al, 2009 [35]	RCT	Coronary artery bypass surgery	232	Daily sessions with a telehealth device (provides strategies to address commonly occurring symptoms experienced after recovery)	A	Discharge	Usual care	1. Physiologic and psychosocial functioning 2. Physical activities 3. Health care use 4. Costs* 5. Symptoms **	6 months after discharge	18	- +
Brink et al, 2007 [29]	Prospec-tive con-trolled trial	Head and neck cancer surgery	184	Electronic health information support system (patients could communicate, get access to information and could be monitored at home)	A	Discharge	Usual care	1. Quality of life	3 months after discharge	17	+
Goldsmith et al, 1999 [36]	RCT	Ambulatory surgery	195	Access to the pain management section of the ambulatory surgery nursing website	A	Before surgery	Website without a pain management section	1. Postoperative pain score (VRS)	Questionnaire was sent home upon discharge	14	+
*Telemonitoring (TM)*											
Ellison et al, 2007 [31]	RCT	Urologic procedures	270	Daily robotic telerounding bedside visits	S	Direct after surgery	Standard daily bedside rounds	1. Postoperative patient morbidity 2. Hospital length of stay 3. Satisfaction with hospitalization	2 weeks after discharge	23	x
**Study characteristics**		**Patients**		**Intervention**			**Control**	**Outcome**			
**ID**	**Design**	**Type of surgery**	**n**	**Description of the intervention**	**Aim** ^**1**^	**Start of the intervention**	**Control group**	**Type of outcome measure**	**Follow-up until**	**Quality score** ^**2**^	**Result** ^***3***^
Keeping et al, 2013 [43]	RCT	Coronary artery bypass graft surgery	182	Telehealth follow-up; audio-video sessions during the first week after discharge	A	Discharge	Usual care	1. Anxiety levels for patients and caregivers 2. Depressive symptoms 3. Health care utilization	3 weeks after discharge	23	+
Ellison et al, 2004 [24]	RCT (three armed)	Urologic procedures	85	1: Daily bedside rounds and an additional telerounding visit once daily. 2: substitution of the bedside round on postoperative day 2 with a telerounding visit	1. A 2. S	First post-operative day	Standard once daily bedside rounds	1. Patient satisfaction with post-operative care	2 weeks after discharge	21	+
Halimi et al, 2008 [28]	Non-inferiority RCT	Pacemaker implantation	379	Enhanced discharge, followed by home monitoring. In the event of a device dysfunction or clinical event, the investigator was notified	S	Discharge	Discharge on the basis of usual care	1. (Major) Adverse events 2.Non major adverse events (NMAE) 3. Duration of hospital stay 4. Quality of life 5. Costs	4 weeks	22	+
Pombo et al, 2013 [42]	RCT	Ambulatory surgery	32	A daily electronic pain diary to assess self-reported pain	A	Direct after surgery	Usual care	1. Pain intensity 2.Compliance	5 days	19	x
Cleeland et al, 2011 [30]	RCT	Thoracic surgery for lung cancer	100	At-home symptom monitoring by automated telephone calls. An alert was forwarded to the clinical team if any a subset of symptoms reached a severity threshold.	A	Discharge	Automated telephone calls without alerts	1. Symptoms 2. Reduction in symptom threshold events 3. Patient satisfaction 4. Satisfaction with the intervention	4 weeks after discharge	15	+
Gandsas et al, 2007 [44]	Retro-spective review	Laparoscopic gastric bypass surgery	376	Robotic bedside rounds in addition to standard bedside rounds	A	Direct after surgery	Standard bedside rounds	1. Duration of hospital stay 2. Readmission rate 3. Costs	?	12	x
Stomberg et al, 2012 [41]	RCT (pilot)	Cholecystec-tomy and hysterectomy	40	Pain assessment by a mobile phone support system	S	The day of surgery	Paper based pain assessment	1. Pains scores 2.Satisfaction with the intervention	6 days	10	-
*Telerehabilitation (TR)*											
Piquares et al, 2013 [45]	Non-inferiority RCT	Total knee arthroplasty	142	Interactive telerehabilitation therapy	S	Discharge	Conventional rehabilitation program	1. Active knee extension and flexion 2. Quadriceps muscle strength 3. Hamstring muscle strength 4. Balance and gait 5. Pain 6. WOMAC	3 months after surgery	24	x
Russel et al, 2011 [46]	Non-inferiority RCT	Total knee arthroplasty	65	Rehabilitation through real-time interaction with a physical therapist across an Internet-based telerehabilitation system	S	One week after discharge	Conventional rehabilitation program	1. WOMAC 2.Patient specific functional Scale 3. Quality of life 4. Timed up-and-go test 5. Pain 6. Satisfaction with the intervention	6 weeks after surgery	23	+
Erikson et al, 2009 [27]	Clinical controlled trial	Shoulder joint replacement	22	Physiotherapy under the supervision of a physiotherapist using videoconferencing	S	Discharge	Physiotherapy in a conventional way	1. Length of hospital stay 2. Number of physiotherapy sessions 3. Pain 4. Range of motion 5. Shoulder function 6. Shoulder condition 7. Health related quality of life	8 weeks after discharge	20	+
Tousignant et al, 2015 [48]	RCT	Total knee arhroplasty	197	In home telerehabilitation group	S	Direct after surgery	Home visit control group	1. Costs	4 months	18	+
Tousignant et al, 2011 [47]	RCT	Total knee arthroplasty	48	Telerehabilitation: videoconferencing with remote-controlled cameras	S	Discharge	Usual care	1. Range of motion 2.Balance 3. Lower body strength 4.Knee function 5. Locomotor performance in walking 6.Functional autonomy 7. Quality of life	3 months after discharge	17	x
Kortke et al, 2006 [26]	Open clinical study	Cardiac surgery	170	Ambulant rehabilitation using telemedicine	S	Discharge	Rehabilitation in a rehabilitation hospital	1. Maximal physical performance 2. Quality of life 3. Complications 4. Costs	12 months after surgery	14	+
**Study characteristics**		**Patients**		**Intervention**			**Control**	**Outcome**			
**ID**	**Design**	**Type of surgery**	**n**	**Description of the intervention**	**Aim** ^**1**^	**Start of the intervention**	**Control group**	**Type of outcome measure**	**Follow-up until**	**Quality score** ^**2**^	**Result** ^***3***^
*Teleconsultation (TC)*											
Zahlman et al, 2002[49]	Histori-cally controlled investi-gation	Cataract surgery	62	One asynchronous and one synchronous teleconsultation with the surgeon to make the decision whether or not to perform surgery	S	Before surgery	Usual care	1. Satisfaction with overall treatment 2.Number of visits to the surgeon's office 3.Duration of consultations	Referral back to the ophtal-mologist	8	+

1: A = the aim of the intervention is to deliver additional care; S = aim of the intervention is to (partly) substitute standard care

2: Quality score classification: good (21–26) fair (14–20); poor (lower than 14)

3: + = significant difference in favour of the intervention group regarding at least one outcome measure;— = significant difference in favour of the control group regarding one outcome measure; x = no significant difference between groups regarding all outcome measures

* Reported in a sub analysis in Young 2012

** Reported in a sub analysis in Zimmerman 2007

*** Reported in Heikinen 2011

**** Reported in Heikinen 2012

#### Participants

Most studies (n = 9) included patients undergoing cardiac surgery, accompanied by seven studies which involved orthopaedic surgery. The indication for surgery was in most studies benign (n = 23); only two studies included patients undergoing surgery because of a malignant indication only [29; 30] and two studies included both [24; 31]. The mean age of the participants varied from 43.2 years [32] to 75.3 years [33]. Most studies included both male and female patients, except for one study [32] which included patients undergoing gynaecological surgery.

#### Type of Interventions

All studies focused on replacing (n = 11) or complementing (n = 15) perioperative usual care by or with some form of care via ICT. One study evaluated both by using two intervention arms [24]. We categorised the methods into four categories according to the main aim of the intervention:

1. An educational or supportive website or device (ESW) to provide information about the surgery and the recovery process, to give positive reinforcement or to provide a tailored rehabilitation program in addition to the usual perioperative care: 12 studies [8; 25; 29; 32–40].
2. Telemonitoring (TM) through electronic questionnaires or by an electronic symptom alert system in or outside the hospital: eight studies [24; 28; 30; 31; 41–44]. In three studies this took place inside the hospital in the form of robotic telerounding and in five studies the telemonitoring took place outside the hospital by electronic symptom questionnaires or vital functioning monitoring. In one study [28] this was part of an enhanced discharge planning intervention and one of these studies also provided audio-video sessions.
3. Telerehabilitation (TR) at home instead of within a rehabilitation center or physiotherapy sessions in a conventional way: six studies [26; 27; 45–48].
4. Teleconsultations (TC) were used instead of a face to face consult with the surgeon in the decision process whether or not to perform surgery: one study [49].

In seven studies the intervention had already started before surgery [8; 25; 32; 36; 37; 40; 49], in five studies the intervention started in hospital after surgery [24; 31; 41; 42; 44], but in most cases the intervention started at or shortly after discharge [26–30; 33–35; 38; 39; 43; 45–48]. As a consequence, most interventions were focused on the period after surgery.

#### Type of Outcome Measures

The outcome measures were classified into three categories:

1. The first category consisted of outcomes regarding the physical component of the postoperative course such as physical functioning, pain and complications. This type of outcome measure was reported in 20 studies [8; 25–39; 41; 42; 45; 46].
2. In the second category outcome measures focusing on the mental component of the postoperative course were defined, such as mental health or anxiety, reported in 14 studies [8; 25–29; 32–35; 38; 40; 43; 47].
3. In the last category general outcome measures regarding the postoperative course were observed (19 studies), such as costs, return to work, satisfaction or length of hospital stay [8; 24–28; 30–33; 35; 40; 41; 43; 44; 46–49].

Seven studies included also reported on outcome measures specific to the type of surgery or intervention, for example cardiovascular risk factor modification adherence, or outcomes measuring the function or condition of the shoulder or knee [26; 27; 30; 34; 45–47]. One study reported on patient knowledge about surgery and recovery [40]. The results of these outcome measures were not considered in this review.

### Risk of Bias in Included Studies

11 studies were judged as having a low risk of bias, 13 studies as medium risk of bias, and three studies as high risk of bias. Five items were scored by a notably low number of studies: if there was made an assumption to blind the patients (n = 2) or the caregivers (n = 5), whether adverse events were being reported (n = 8), if the study had sufficient power to detect a clinically important effect (n = 9) and if compliance with the intervention was reliable (n = 10) (S1 Table).

### Outcomes

17 studies (63.0%) reported a significant effect in favour of the intervention group regarding at least one of the reported outcome measures (Table 1). Eight studies (29.6%) reported no significant differences between the groups. Two studies (7.4%) found an effect in favour of the control group, but one of these studies also found a positive effect with regards to the intervention group relating to one outcome measure.

In total, 12 studies evaluated an ESW intervention. In eight studies (66.7%) a significant difference in favour of the intervention group was observed. In the eight studies in which a TM intervention was evaluated, a significantly positive effect was found in four studies (50.0%). Moreover four out of six studies (66.7%) reported a positive effect of a TR intervention. The only study which evaluated a TC intervention found a significant difference with regards to the intervention group.

11 out of the 15 studies that evaluated an intervention in addition to usual care found a significant difference between groups in favour of the intervention group (73.3%). Of the 11 studies that evaluated an intervention which substituted the usual care, six found a positive effect (54.5%).

Table 2 shows the overall results of the positive or negative effects for the different types of reported outcome measures.

**Table 2:** Results regarding the different types of outcome measures.

	Number of studies reporting this outcome measure	Significant effect in favour of the intervention group	Significant effect in favour of the control group	No significant difference
**Physical**				
Physical functioning	**10**	**6**	**0**	**4**
Physical activities	**2**	**0**	**1**	**1**
Pain	**9**	**3**	**1**	**5**
Symptoms	**5**	**1**	**0**	**4**
Complications	**3**	**1**	**0**	**2**
**Mental**				
Psychosocial functioning	**9**	**4**	**0**	**5**
Anxiety	**3**	**1**	**0**	**2**
Depression	**1**	**0**	**0**	**1**
Emotions	**1**	**0**	**0**	**1**
Self-efficacy	**1**	**1**	**0**	**0**
Autonomy	**1**	**0**	**0**	**1**
**General**				
General quality of life	3	1	0	2
Satisfaction	6	4	0	2
Return to work	1	1	0	0
Length of recovery	1	0	0	1
Health care use	6	2	0	4
Length of hospital stay	4	1	0	3
Costs	6	2	0	4

### 1. Outcomes Regarding the Physical Component of the Postoperative Course.

#### 1.1 Physical Functioning

In Table 3, the study results of the 10 studies reporting physical functioning scores are presented. Regarding physical functioning, six studies showed significant changes between groups in favour of the intervention group [27; 29; 32–34; 46]. Four of these studies used the SF-36 as a measuring instrument [27; 32–34] Two studies used questionnaires. As well as this one study used a self-developed quality of life questionnaire with five physical functioning subscales [29]. Of these five subscales, the physical self-efficacy subscale showed a significant difference 6 weeks and 3 months after surgery, whereas the general physical complaints and perceived abilities in swallowing and food intake only showed a significant difference 6 weeks after surgery. One study reported a significant difference in the absolute mean change of the Patient-Specific Functional Scale [46]. Pertaining to these six studies, four were rated as being of medium risk of bias and two of low risk. All of these studies (mainly) focused on the period after discharge, with four studies evaluating an ESW intervention. Moreover, only one study started prior to surgery [32], the other five studies started at the moment of discharge or one week afterwards.

**Table 3:** Studies reporting physical functioning scores.

ID	Measuring instrument	Measuring moments	Effect^1^	Details of the effect	Note
*Education or supportive website or device (ESW)*					
**Barnason et al, 2003 [34]**	4 physical functioning subscales of the SF36	Prior to discharge—6 wks—3 months	+	2 subscales showed significant higher adjusted mean scores in the intervention group (physical functioning and general health functioning)	Subsample of an unpublished study of Barnason.
**Barnason et al, 2006 [33]**	4 physical functioning subscales of the SF36	Prior to discharge—6 wks—3 months	+	1 subscale showed significant higher adjusted mean scores in the intervention group (general health functioning)	
**Brink et al, 2007 [29]**	5 physical functioning subscales of a quality of life questionnaire	Prior to discharge—6 wks—3 months	+	2 subscales showed significant improved scores 6 weeks after surgery in the intervention group (perceived abilities in swallowing and food intake and general physical complaints) 1 subscale showed significant improved scores 6 weeks and 3 months after surgery in the intervention group (physical self-efficacy)	
**Vonk et al, 2014 [32]**	4 physical functioning subscales of the SF36	Prior to surgery—2 wks—6 wks—12 wks—26 wks	+	Significantly more improvement in the physical health part of the SF-36 in the intervention group	
**Barnason et al, 2009 [35]**	3 physical functioning subscales of the SF36	Prior to dis-charge—3 wks—6 wks—3 months	x	No significant differences for any of the three subscales	Sub analyses of patients with high disease burden (n = 55) [50] and of female patients (n = 40) [51] did not show significant differences. No differences in physical functioning scores were found between men and women [52].
**Miller et al, 2007 [38]**	3 physical functioning subscales of the SF36	Prior to discharge—6 wks—3 months	x	No significant differences for any of the three subscales	The intervention group had a greater improvement on all of the 3 subscales between baseline and 3 months than the control group.
*Telemonitoring (TM)*					
**Halimi et al, 2008 [28]**	4 physical functioning subscales of the SF36	1 month after inclusion	x	No significant difference between groups for the mean score of the physical health part of the SF-36	
*Telerehabilitation (TR)*					
**Erikson et al, 2009 [27]**	4 physical functioning subscales of the SF36	Week before surgery—8 wks	+	The intervention group improved significantly more between baseline and follow-up on 1 subscale (decrease in pain)	
**Russel et al, 2011 [46]**	Patient-Specific functional Scale	One week after discharge– 6 wks	+	Significantly more improvement in the intervention group for the total score of the patient-specific functional scale	
**Kortke et al, 2006 [26]**	4 physical functioning subscales of the SF36	Prior to surgery—6 months—12 months	x	No between group comparison available.	The intervention group significantly improved regarding all 4 subscales 6 months after surgery, the control group only regarding to 2 subscales

1: between group comparison

+ = significant effect regarding one or more subscales in favour of the intervention group

- = significant effect regarding one or more subscales in favour of the control group

x = no significant differences between groups

A particular study (n = 170) with a medium risk of bias reported no difference in effect between groups for physical functioning, however they reported an increase of scores in both groups compared to baseline values, which was only significant regarding all subscales in the intervention group (TR) [26]. The remaining three studies showed no difference in effect between groups for any of the subscales [28; 35; 38]. One of these was the large, medium risk of bias study of Barnason 2009 [35], in contrast to the two earlier studies of Barnason [33; 34] in which a positive effect of the intervention was reported. This study from 2009 was very similar to the previous two studies from these researchers, however it consisted of a bigger sample and a longer follow-up duration.

#### 1.2 Physical Activities

Two ESW studies measured physical activities using an activity diary and RT3 accelerometer. One large study (n = 232, medium risk of bias) reported a significant change in estimated energy expenditure measured by the RT3 accelerometer; the control group showed higher scores three weeks after surgery when compared to the intervention group [35]. One medium risk of bias study (n = 49) reported no difference in effect for this outcome measure between groups [38].

#### 1.3 Pain-related Outcome Measures

Nine studies measured pain scores (Table 4), of which three reported a positive effect in the postoperative pain score for the intervention group. These studies all vary based on the type of surgery, type of intervention and duration and timing of the interventions. Five studies presented no significant differences in pain scores between groups [25; 37; 42; 45; 46]. Two of these were non-inferiority studies [45; 46]. One study (n = 40, high risk of bias) reported significantly higher pain levels in the intervention group [41]. In this study the assessment of postoperative pain was evaluated. The intervention group (TM) responded by mobile phones and the control group by paper-based questionnaires.

**Table 4:** Studies reporting pain scores.

ID	Type of outcome measure	Measuring instrument	Measuring moments	Effect ^1^	Details of the effect	
					Mean Intervention group (SD or range)	Mean Control group (SD or range)
*Education or supportive website or device (ESW)*						
**Goldsmith et**	Postoperative	5-point Verbal	- Discharge	+	- NR	- NR
**al, 1999 [36]**	pain score	Rating Scale	- The night after surgery		- NR	- NR
			- Day after surgery		- NR	- NR
**Vonk**	Pain intensity	VAS	- Before surgery	+	- NR	- NR
**et al, 2014 [32]**			- 2 weeks after surgery		- NR	- NR
			- 6 weeks after surgery		- NR	- NR
			- 12 weeks after surgery		- NR	- NR
			- 26 weeks after surgery		- 1.92 (0.41)	- 3.52 (0.58)
**Martorella et**	Pain intensity	Numerical	- Day 1—Day 2—Day 3—Day 7 after	x	NR	NR
**al, 2012 [37]**		rating scale	surgery			
		(0–10)				
**Neary**	Postoperative	VAS (0–10)	- 24h following surgery	x	- 3.45 (2.7)	- 3.38 (2.7)
**et al, 2010 [25]**	pain score					
*Telemonitoring (TM)*						
**Pombo et al,**	Pain intensity	Numerical	- 24h after surgery	x	- 2.1 (1.06)	- 2.3 (1.06)
**2013 [42]**		rating scale	- 5 day after surgery		- 1.86 (1.86)	- 2 (0)
		(0–10)				
**Stomberg et al,**	Pain score	Numerical	Every four hours from the day of surgery	-	NR	NR
**2012 [41]**		rating scale	until 6 days after surgery			
		(0–100)				
*Telerehabilitation (TR)*						
**Erikson**	Median	VAS (0–10)	- Between the week before surgery and 8	+	- 7 (3–10)	- 2 (-1-5)
**et al, 2009 [27]**	improvement in		weeks after discharge			
	pain					
**Piquares et al,**	Mean change in	VAS	- Between baseline and completing	x	- 0.69 (1.44)	- 0.61 (1.87)
**2013 [45]**	pain score		rehabilitation			
			- Between baseline and 3 months after		- 1.79 (2.45)	- 2.3 (2.03)
			surgery			
**Russel**	Mean change in	VAS	- Between baseline and 6 weeks after	x	- 3.07 (1.55)	- 3.29 (1.31)
**et al, 2011 [46]**	pain score		surgery			

1: between group comparison

+ = significant effect in favour of the intervention group

- = significant effect regarding in favour of the control group

x = no significant differences between groups

Two low risk of bias, ESW studies measured analgesic consumption or requirements [25; 37]. One such study (n = 64) reported no change in effect between the intervention and the usual care group [25]. The second study (n = 60) observed significantly more use of opioid medication in the intervention group than in the control group [37]. However, regarding pain interference with daily activities, a positive effect of the intervention was found for pain interference with breathing/coughing 3 days after surgery. A similar positive influence was found for the intervention group in pain interference on appetite on day 7 after surgery compared to the control group.

#### 1.4 Postoperative Symptoms or Problems

Five studies reported problems or symptoms in the postoperative course, which were not rated as complications [8; 30; 33; 39; 51]. In a particular study (sub analyses on female patients, n = 45, low risk of bias) a positive effect was observed for one out of ten symptoms; patients who received daily sessions with a telehealth device (ESW intervention) reported significantly lower fatigue scores than patients who received usual care six weeks after CABG surgery [51]. The other studies (three ESW interventions and one TM intervention) reported no significant differences in symptom scores between groups.

#### 1.5 Complications

Three studies reported complications during follow up. No studies found a higher instance of complications in the intervention group. One study (n = 170, medium risk of bias) reported more difficulties in the control group than in the intervention group (TR) during follow up [26]. Two other studies [28; 31] reported no differences between groups. One of these studies was a non-inferiority study in which complications were the primary outcome measure [28].

### 2. Outcomes Related to the Psychosocial or Mental Component of the Recovery Process.

#### 2.1 Psychosocial Functioning

There were nine studies that described the psychosocial functioning subscales of quality-of-life questionnaires (Table 5). Of these, four found significant differences between groups that favoured the intervention group with regards to one or more subscales [27; 29; 32; 34]. Two of these were small studies (n = 35 and n = 22) with a medium risk of bias and reported a positive effect of the intervention on the vitality subscales of the SF-36 [27; 34]. In one such study there was a positive effect of the intervention on the Mental Health subscale [34]. The third study which reported an effect was a study with 184 participants undergoing head and neck cancer surgery and with a medium risk of bias. In this study a positive effect was reported on 2 out of 17 mental health subscales (anxiety and fear related to head and neck problems) 6 weeks after discharge [29]. The fourth study which reported an effect in psychosocial functioning was a low risk of bias study with 215 participants [32]. A positive effect of the intervention (ESW) was reported for the mental component of the SF-36.

**Table 5:** Studies reporting psychosocial functioning scores.

ID	Measuring instrument	Measuring moments	Effect ^1^	Details of the effect	Note
*Education or supportive website or device (ESW)*					
**Barnason et al, 2003 [34]**	4 psychosocial functioning subscales of the SF36	Across time	+	2 subscales showed significant higher adjusted mean scores in the intervention group (mental health and vitality)	No significant differences for the other two subscales
**Brink et al, 2007 [29]**	17 psychosocial functioning subscales of a self-developed quality of life questionnaire	Prior to discharge—6 wks—3 months	+	2 subscales showed significant improved scores 6 weeks after surgery in the intervention group (state of anxiety and fear related to specific head and neck problems)	3 months after surgery there were no significant differences between groups
**Vonk et al, 2014 [32]**	4 psychosocial functioning subscales of the SF36	Prior to surgery—2 wks—6 wks—12 wks—26 week	+	Significantly more improvement in the mental health part of the SF-36 in the intervention group	
**Barnason et al, 2006 [33]**	4 psychosocial functioning subscales of the SF36	Across time	x	No significant differences for any of the four subscales	
**Barnason et al, 2009 [35]**	3 psychosocial functioning subscales of the SF36	Across time	x	No significant differences for any of the three subscales	Sub analyses of patients with high disease burden (n = 55) [50], did not show significant differences
**Miller et al, 2007 [38]**	3 psychosocial functioning subscales of the SF36	Prior to discharge—6 wks—3 months	x	No significant difference between groups for any of the three subscales	
*Telemonitoring (TM)*					
**Halimi et al, 2008 [28]**	4 psychosocial functioning subscales of the SF36	1 month	x	No significant difference between groups in the mental health part of the SF-36	
*Telerehabilitation (TR)*					
**Erikson et al, 2009 [27]**	4 psychosocial functioning subscales of the SF36	Week before surgery—8 wks	+	The intervention group improved significantly more than the control group on one subscale (vitality)	
**Kortke et al, 2006 [26]**	4 psychosocial functioning subscales of the SF36	6 months	x	No between group comparison reported.	The intervention group significantly improved with regard to all 4 subscales 6 months after surgery, the control group with regard to none of the subscales

1: between group comparison

+ = significant effect in favour of the intervention group

- = significant effect regarding in favour of the control group

x = no significant differences between groups

#### 2.2 Anxiety, Depression and Emotions

In total, four studies measured mental health recovery with instruments other than the quality-of-life questionnaires. There were also two studies with a low risk of bias that measured anxiety with the S-STAI or the HADS respectively [25; 43]. No differences in anxiety scores at follow up were measured. One study measured anxiety about recovery after an ESW intervention with a self-developed questionnaire [40]. Participants from the intervention group were significantly less anxious about their recovery. Depression was measured with the CASD-10, which also did not show any significant differences between groups [43]. Postoperative emotion scores were measured in a low risk of bias study with 147 participants undergoing orthopaedic surgery [53]. No effect on emotions was measured between the ESW intervention and the control group.

#### 2.3 Self-efficacy and Autonomy

We found one study (n = 48, medium risk of bias) which measured functional autonomy in patients undergoing total knee arthroplasty using the SMAF [47; 47; 48], and reported no difference in effect between the two groups after two months. Self-efficacy was measured in a small study (n = 35, medium risk of bias) by the Barnason Efficacy Expectation scale 6 weeks and 3 months after CABG surgery [34]. The intervention group (ESW) reported significantly higher adjusted mean scores across time compared to the usual care groups.

### 3. General Outcome Measures in Relation to the Recovery Process.

#### 3.1 General Quality of Life

In total 11 studies used quality of life measurements. Nine of them were represented earlier in this review because they reported separate physical or psychosocial outcomes. However, two relatively small studies measured quality of life total scores for patients undergoing total knee arthroplasty, and found no difference in effect [46; 47]. Furthermore, one study (n = 215, low risk of bias) also measured the total scores of the SF-36, next to the separate physical and mental component scores, and reported a positive effect of the intervention (ESW) [32].

#### 3.2 Satisfaction

Four out of six studies that compared overall satisfaction with the treatment between groups found a significant difference in effect in favour of the intervention group [24; 30; 40; 49]. Five studies [25; 30; 40; 41; 46] evaluated patient satisfaction, with a particular focus on the intervention, without measuring the control group. They all reported that patients were very satisfied.

#### 3.3 Length of Recovery

In only one study (n = 215, low risk of bias) the return to work rate was compared between both groups [32]. In this study a significant difference in return to work of nine days was reported due to the intervention (ESW). They also measured the effect of the intervention by a validated recovery outcome follow-up (RI-10). No difference between both groups was measured.

#### 3.4 Health Care Usage

In all, six studies measured health care usage in the postoperative period, but there were some important differences in the source of health care use the studies evaluated. Four studies measured the number of visits to the physician. However, two medium risk of bias studies (n = 50 and n = 232) reported no significant differences between groups [33; 35]. Two studies reported significantly more visits in the control group [43; 49]. In one study (n = 62, high risk of bias) this was not a surprising finding since the intervention in this study consisted of a teleconsult instead of a regular hospital visit (TC intervention) [49]. One study measured the number of physiotherapy sessions during a TR intervention [27]. The telemedicine group received a greater number of treatments compared to the control group, but it was not described whether or not this difference was significantly or clinically relevant. The three studies which reported the number of emergency department visits found no significant differences between groups [33; 35; 43], nor did the four studies which reported the number of re-admissions in the hospital [33; 35; 43; 44].

#### 3.5 Length of Hospital Stay

There were four studies which measured hospital length of stay [27; 28; 31; 44]. Only one of these studies (n = 379, medium risk of bias) reported a positive effect using a home monitoring program (TM intervention) after a pacemaker implantation on hospital length of stay (3.2 days SD 3.2 vs 4.8 days SD 3.7) [28].

#### 3.6 Costs

Five out of six studies reported on costs related to direct and indirect health care costs [26; 28; 48; 54; 55]. The majority (n = 4) included the extra costs for the intervention [26; 48; 54; 55]. Only one trial reported the cost-effectiveness of the intervention calculating the ICER related to the effect on physical activity [55]. There was also a trial which only reported the estimated cost savings based on the length of stay in hospital [44]. Only two studies reported a positive effect in costs [26; 48]. For one such study the effect depended on the travel distance for the patient between their residence and the hospital. For the other three studies no difference in costs were measured between the two groups [48]. All were large studies (at least 147 participants) but with a high [44] or medium risk of bias [26; 28; 48; 54; 55].

## Discussion

### Main Findings

In this systematic review we evaluated the effect of complementing or substituting care by perioperative e-health interventions on the postoperative course based on the results of 27 included studies. There was a large diversity in studies regarding to type of patients, interventions and outcome measures. 25 studies (92.6%) reported at least an equal (n = 8) or positive effect (n = 17) of the e-health intervention compared to usual care. In two studies (7.4%) also a positive effect was observed for all outcomes in favour of the control group. Most studies evaluated an ESW intervention. There were no considerable differences in the effectiveness between the different types of e-health interventions. No association was found between the aim of the intervention (addition of care or substitution of care) and its effectiveness. The majority of the studies (n = 9) included patients undergoing cardiac surgery. Of these, seven studies (77.7%) found a positive effect with regards to the intervention group concerning one or more of the noted outcome measures. Seven studies included patients undergoing orthopedic surgery. Three of these studies (42.9%) reported a positive effect. However, these populations are very troublesome to compare as there was a wide diversity in the type of e-health interventions which were evaluated in both groups.

We categorised the outcome measures which were reported in the different studies into physical, mental and general outcome measures. Overall the results in these outcomes measures were comparable which suggests that there were no specific differences in the effect of e-health interventions on the different types of postoperative outcome measures.

As well as categorising outcome measures into physical, mental and general, another categorisation could have been made: outcome measures focusing on the additional value of e-health interventions on patients’ wellbeing (such as physical or mental functioning, pain, satisfaction) and outcome measures focusing on the efficiency of e-health interventions (such as health care use and costs). The second category type of outcome measures was notably less used in the selected studies (n = 11). Of these, six studies reported on costs. These studies were all relatively large, but were all considered to have a medium risk of bias. Two studies observed a positive effect in costs [26; 48].

### Strengths and Limitations

This is the first systematic review published on the usage of e-health in the perioperative care. Another strength of this review is methodological quality, ensured by following the Prisma guidelines [18] for systematic reviews. We conducted a very broad literature search and carefully evaluated the different type of search terms which could possibly be used. Due to the wide range of inclusion criteria, we were able to report a broad overview of the potential health benefits of the application of e-health interventions for various types of perioperative care.

A potential limitation may be the exclusion of four non-English publications that could have been relevant within the scope of our review. Another potential limitation could be not using all search terms within our search strategy because of the enormous amount of literature by using the extra term ‘surgery’. This yielded another 4405 extra titles. After screening the first 500 hits, bringing only two extra relevant hits not retrieved in our initial search, we decided to use the cross-references and related citations of the included studies instead. However, we cannot exclude that we missed studies because of this procedure. Most of the comprised studies were judged as being of a medium risk of bias. In this assessment, five items were scored by a notably low number of studies: if an assumption was made to blind the patients or the caregivers, whether adverse events were being reported, if compliance with the intervention was reliable and if the study had sufficient power to detect a clinically important effect. We considered all five items to be important risk factors for introducing bias Although, we understand that blinding of the patients and caregivers is difficult in this type of studies, measuring the compliance and adverse events should be an integral part for this type of research. In addition, the fact that only nine studies performed a power calculation and included enough patients, requires to interpret the results of this review with caution.

Another limitation is that it was not possible to conduct a meta-analysis due to heterogeneity in terms of type of surgery, type of intervention and the follow-up period. Finally, we did not report the disease or surgery-specific health outcomes as the aim of our review was to give a broad overview of the implication of e-health interventions in general perioperative care. It could however be that this may have under–or overestimated the effect of the e-health interventions on recovery.

### Comparison with Other Studies

Our results are in line with the results of various systematic reviews focusing on the effects of complementing or substituting care by e-health in general medical care. Flodgren et al, 2015 published a Cochrane systematic review about the effectiveness of e-health on professional practice and health care outcomes [56]. They concluded that the use of e-health leads to, minimally, similar health outcomes as usual care and may probably improve health care. Ekeland et al, 2010 evaluated the effect of telemedicine interventions in general medical care in a systematic review [57]. They included 80 systematic reviews, of which 21 studies concluded that e-health was effective and in 18 studies the evidence was limited and inconsistent. These results are in line with our review in which 17 out of 27 studies (63%) reported a positive effect for one or more outcome measures. In our review, only limited study data were included which related to the cost-effectiveness of e-health. In line, de la Torre-Dıez et al. 2015, published a review about the cost-utility and cost-effectiveness for telemedicine in general medical care and concluded there is a lack of good quality, cost-effective studies. [58] Eland de Kok et al. 2011, systematically reviewed the effects of e-health on care for chronically ill patients [59]. They concluded that the usage of e-health leads to moderately positive effects on primary health outcomes, but again concluded a lack of cost-effectiveness studies. [56]

### Conclusion and Clinical Relevance

Based on this systematic review we conclude that e-health interventions with the aim to complement or substitute perioperative care by educational websites, telemonitoring interventions, telerehabilitation programs and teleconsultations probably improves clinical patient outcomes compared with conservative face to face perioperative care for patients who have undergone various forms of surgery. There is, however, a lack of good quality (cost)-effective studies included in this review, with only a limited proportion of studies reporting they have performed a power calculation or have measured the compliance, or report about the occurrence of adverse events. For the future, we strongly recommend high quality cost-effective studies to provide more evidence for practitioners and policymakers whether or not they should implement e-health interventions in perioperative care.

## Supporting Information

S1 Checklist PRISMA-2009-Checklist. (DOC)

S1 Form Risk of bias scoring form—Downs and Black. (DOCX)

S1 Table Risk of bias of the individual studies. (XLSX)

S1 Text Search strategies 2 dec 2015. (DOCX)

## References

[1] Duplaga M (2013) The acceptance of e-health solutions among patients with chronic respiratory conditions. Telemed J E Health 19:683–691 10.1089/tmj.2012.0306 23734700 PMC3757530

[2] Fairbrother P, Ure J, Hanley J, McCloughan L, Denvir M, Sheikh A et al (2014) Telemonitoring for chronic heart failure: the views of patients and healthcare professionals—a qualitative study. J Clin Nurs 23:132–144 10.1111/jocn.12137 23451899

[3] Tang C, Lorenzi N, Harle CA, Zhou X, Chen Y (2016) Interactive systems for patient-centered care to enhance patient engagement. J Am Med Inform Assoc 23:2–4 10.1093/jamia/ocv198 26912537 PMC7814929

[4] Barello S, Triberti S, Graffigna G, Libreri C, Serino S, Hibbard J et al (2015) eHealth for Patient Engagement: A Systematic Review. Front Psychol 6:2013 10.3389/fpsyg.2015.02013 26779108 PMC4705444

[5] Cook DJ, Manning DM, Holland DE, Prinsen SK, Rudzik SD, Roger VL et al (2013) Patient engagement and reported outcomes in surgical recovery: effectiveness of an e-health platform. J Am Coll Surg 217:648–655 10.1016/j.jamcollsurg.2013.05.003 23891066

[6] Bouwsma EV, Vonk NA, Szlavik Z, Brolmann HA, Emanuel MH, Lips JP et al (2014) Process evaluation of a multidisciplinary care program for patients undergoing gynaecological surgery. J Occup Rehabil 24:425–438 10.1007/s10926-013-9475-4 24057871 PMC4118044

[7] Eaton L, Walsh C, Magnuson T, Schweitzer M, Lidor A, Nguyen H et al (2012) On-line bariatric surgery information session as effective as in-person information session. Surg Obes Relat Dis 8:225–229 10.1016/j.soard.2011.10.015 22178563

[8] Heikkinen K, Leino-Kilpi H, Vahlberg T, SalanterÃ¤ S (2012) Ambulatory orthopaedic surgery patientsâ€™ symptoms with two different patient education methods. International Journal of Orthopaedic & Trauma Nursing 16:13–20

[9] Hering K, Harvan J, D'Angelo M, Jasinski D (2005) The use of a computer website prior to scheduled surgery (a pilot study): impact on patient information, acquisition, anxiety level, and overall satisfaction with anesthesia care. AANA Journal 73:29–33 15727280

[10] D'Haese P-F, Pallavaram S, Li R, Remple MS, Kao C, Neimat JS et al (2012) CranialVault and its CRAVE tools: A clinical computer assistance system for deep brain stimulation (DBS) therapy. Med Image Anal 16:744–753 10.1016/j.media.2010.07.009 20732828 PMC3021628

[11] de VH, Watson MO, Salvado O, Passenger JD (2011) Progress in virtual reality simulators for surgical training and certification. Med J Aust 194:S38–S40 21401487 10.5694/j.1326-5377.2011.tb02942.x

[12] Palombo D, Mugnai D, Mambrini S, Robaldo A, Rousas N, Mazzei R et al (2009) Role of Interactive Home Telemedicine for Early and Protected Discharge 1 Day after Carotid Endarterectomy. Ann Vasc Surg 23:76–80 10.1016/j.avsg.2008.06.013 18809294

[13] Sharareh B, Schwarzkopf R (2014) Effectiveness of telemedical applications in postoperative follow-up after total joint arthroplasty. J Arthroplasty 29:918–922 10.1016/j.arth.2013.09.019 24342278

[14] Bisgaard T, Stockel M, Klarskov B, Kehlet H, Rosenberg J (2004) Prospective analysis of convalescence and early pain after uncomplicated laparoscopic fundoplication. Br J Surg 91:1473–1478 15386321 10.1002/bjs.4720

[15] Tran TT, Kaneva P, Mayo NE, Fried GM, Feldman LS (2014) Short-stay surgery: what really happens after discharge? Surgery 156:20–27 10.1016/j.surg.2014.03.024 24856316

[16] Callesen T, Klarskov B, Bech K, Kehlet H (1999) Short convalescence after inguinal herniorrhaphy with standardised recommendations: duration and reasons for delayed return to work. Eur J Surg 165:236–241 10231657 10.1080/110241599750007108

[17] Vonk NA, Anema JR, Louwerse MD, Heymans MW, van MW, Brolmann HA et al (2014) Prediction of time to return to work after gynaecological surgery: a prospective cohort study in the Netherlands. BJOG 121:487–497 10.1111/1471-0528.12494 24245993

[18] Liberati A, Altman DG, Tetzlaff J, Mulrow C, Gotzsche PC, Ioannidis JP et al (2009) The PRISMA statement for reporting systematic reviews and meta-analyses of studies that evaluate health care interventions: explanation and elaboration. PLoS Med 6:e1000100 10.1371/journal.pmed.1000100 19621070 PMC2707010

[19] Pagliari C, Sloan D, Gregor P, Sullivan F, Detmer D, Kahan JP et al (2005) What is eHealth (4): a scoping exercise to map the field. J Med Internet Res 7:e9 15829481 10.2196/jmir.7.1.e9 PMC1550637

[20] Cochrane Consumers and Communication Review Groups: Tools and guides for review authors. Data extraction template. 2013. Available: http://cccrg.cochrane.org/author-resources. Accessed 22 April 2014. (2015)

[21] Downs SH, Black N (1998) The feasibility of creating a checklist for the assessment of the methodological quality both of randomised and non-randomised studies of health care interventions. J Epidemiol Community Health 52:377–384 9764259 10.1136/jech.52.6.377 PMC1756728

[22] Eng JJ, Teasell R, Miller WC, Wolfe DL, Townson AF, Aubut JA et al (2007) Spinal Cord Injury Rehabilitation Evidence: Methods of the SCIRE Systematic Review. Top Spinal Cord Inj Rehabil 13:1–10 22767989 10.1310/sci1301-1 PMC3389040

[23] Mahmud N, Schonstein E, Schaafsma F, Lehtola MM, Fassier JB, Reneman MF et al (2010) Pre-employment examinations for preventing occupational injury and disease in workers. Cochrane Database Syst Rev CD008881 10.1002/14651858.CD008881 21154401

[24] Ellison LM, Pinto PA, Kim F, Ong AM, Patriciu A, Stoianovici D et al (2004) Telerounding and patient satisfaction after surgery. J Am Coll Surg 199:523–530 15454133 10.1016/j.jamcollsurg.2004.06.022

[25] Neary PM, Sung R, Corrigan M, O'Donovan M, Cahill RA, Redmond HP (2010) The benefits of an interactive, individualized online patient pathway for patients undergoing minimally invasive radioguided parathyroidectomy: a prospective, double-blinded, randomized clinical trial. Surg Innov 17:236–241 10.1177/1553350610374603 20647234

[26] Kortke H, Stromeyer H, Zittermann A, Buhr N, Zimmermann E, Wienecke E et al (2006) New East-Westfalian Postoperative Therapy Concept: a telemedicine guide for the study of ambulatory rehabilitation of patients after cardiac surgery. Telemed J E Health 12:475–483 16942420 10.1089/tmj.2006.12.475

[27] Eriksson L, Lindstrom B, Gard G, Lysholm J (2009) Physiotherapy at a distance: A controlled study of rehabilitation at home after a shoulder joint operation. J Telemed Telecare 15:215–220 10.1258/jtt.2009.081003 19590025

[28] Halimi F, Clémenty J, Attuel P, Dessenne X, Amara W (2008) Optimized post-operative surveillance of permanent pacemakers by home monitoring: the OEDIPE trial. Europace 10:1392–1399 10.1093/europace/eun250 18775878 PMC2639329

[29] Brink JL, Moorman PW, Boer MF, Hop WC, Pruyn JF, Verwoerd CD et al (2007) Impact on quality of life of a telemedicine system supporting head and neck cancer patients: a controlled trial during the postoperative period at home. Journal of the American Medical Informatics Association: JAMIA 14:198–205 17213498 10.1197/jamia.M2199 PMC2213461

[30] Cleeland CS, Wang XS, Shi Q, Mendoza TR, Wright SL, Berry MD et al (2011) Automated symptom alerts reduce postoperative symptom severity after cancer surgery: a randomized controlled clinical trial. Journal of Clinical Oncology 29:994–1000 10.1200/JCO.2010.29.8315 21282546 PMC3068055

[31] Ellison LM, Nguyen M, Fabrizio MD, Soh A, Permpongkosol S, Kavoussi LR (2007) Postoperative robotic telerounding: a multicenter randomized assessment of patient outcomes and satisfaction. Archives of surgery (Chicago, Ill: 1960) 142:1177–1181 10.1001/archsurg.142.12.1177 18086984

[32] Vonk Noordegraaf A, Anema J, van Mechelen W, Knol D, van Baal W, van Kesteren P et al (2014) A personalised eHealth programme reduces the duration until return to work after gynaecological surgery: results of a multicentre randomised trial. BJOG 121:1127–1136 10.1111/1471-0528.12661 24511914

[33] Barnason S, Zimmerman L, Nieveen J, Hertzog M (2006) Impact of telehealth intervention to augment home health care on functional and recovery outcomes of elderly patients undergoing coronary artery bypass grafting. Heart & Lung 35:225–233 16863894 10.1016/j.hrtlng.2005.10.003

[34] Barnason S, Zimmerman L, Nieveen J, Schmaderer M, Carranza B, Reilly S (2003) Impact of a home communication intervention for coronary artery bypass graft patients with ischemic heart failure on self-efficacy, coronary disease risk factor modification, and functioning. Heart Lung 32:147–158 12827099 10.1016/s0147-9563(03)00036-0

[35] Barnason S, Zimmerman L, Nieveen J, Schulz P, Miller C, Hertzog M et al (2009) Influence of a symptom management telehealth intervention on older adults' early recovery outcomes after coronary artery bypass surgery. Heart & Lung 38:364–376 19755186 10.1016/j.hrtlng.2009.01.005 PMC2900787

[36] Goldsmith DM, Safran C (1999) Using the Web to reduce postoperative pain following ambulatory surgery. Proc AMIA Symp780-784 PMC2232814 10566466

[37] Martorella G, Côté J, Racine M, Choinière M (2012) Web-based nursing intervention for self-management of pain after cardiac surgery: pilot randomized controlled trial. J Med Internet Res 14:e177 10.2196/jmir.2070 23241361 PMC3799541

[38] Miller C, Zimmerman L, Barnason S, Nieveen J (2007) Impact of an early recovery management intervention on functioning in postoperative coronary artery bypass patients with diabetes. Heart Lung 36:418–430 18005803 10.1016/j.hrtlng.2007.02.011

[39] Zimmerman L, Barnason S, Nieveen J, Schmaderer M (2004) Symptom management intervention in elderly coronary artery bypass graft patients. Outcomes Manag 8:5–12 14740578

[40] Yin B, Goldsmith L, Gambardella R (2015) Web-Based Education Prior to Knee Arthroscopy Enhances Informed Consent and Patient Knowledge Recall: A Prospective, Randomized Controlled Study. J Bone Joint Surg Am 97:964–971 10.2106/JBJS.N.01174 26085529

[41] Stomberg MW, Platon B, Widen A, Wallner I, Karlsson O (2012) Health information: what can mobile phone assessments add? Perspect Health Inf Manag 9:1–10 PMC3510647 23209453

[42] Pombo N, Araujo P, Viana J, da Costa MD (2014) Evaluation of a ubiquitous and interoperable computerised system for remote monitoring of ambulatory post-operative pain: A randomised controlled trial. Technol Health Care 22:63–75 10.3233/THC-130774 24398815

[43] Keeping-Burke L, Purden M, Frasure-Smith N, Cossette S, McCarthy F, Amsel R (2013) Bridging the transition from hospital to home: effects of the VITAL telehealth program on recovery for CABG surgery patients and their caregivers. Research in nursing & health 36:540–553 24242195 10.1002/nur.21571

[44] Gandsas A, Parekh M, Bleech MM, Tong DA (2007) Robotic telepresence: profit analysis in reducing length of stay after laparoscopic gastric bypass. J Am Coll Surg 205:72–77 17617335 10.1016/j.jamcollsurg.2007.01.070

[45] Piqueras M, Marco E, Coll M, Escalada F, Ballester A, Cinca C et al (2013) Effectiveness of an interactive virtual telerehabilitation system in patients after total knee arthoplasty: a randomized controlled trial. J Rehabil Med 45:392–396 10.2340/16501977-1119 23474735

[46] Russell TG, Buttrum P, Wootton R, Jull GA (2011) Internet-based outpatient telerehabilitation for patients following total knee arthroplasty: a randomized controlled trial. Journal of Bone & Joint Surgery, American Volume 93:113–120 21248209 10.2106/JBJS.I.01375

[47] Tousignant M, Moffet H, Boissy P, Corriveau H, Cabana F, Marquis F (2011) A randomized controlled trial of home telerehabilitation for post-knee arthroplasty. J Telemed Telecare 17:195–198 10.1258/jtt.2010.100602 21398389

[48] Tousignant M, Moffet H, Nadeau S, Merette C, Boissy P, Corriveau H et al (2015) Cost analysis of in-home telerehabilitation for post-knee arthroplasty. J Med Internet Res 17:e83 10.2196/jmir.3844 25840501 PMC4397389

[49] Zahlmann G, Mertz M, Fabian E, Strobl H, Holle R, Kaatz H et al (2002) Perioperative cataract OP management by means of teleconsultation. Graefe's Arch Clin Exp Ophthalmol 240:17–20 11954775 10.1007/s00417-001-0396-0

[50] Barnason S, Zimmerman L, Schulz P, Tu C (2009) Influence of an early recovery telehealth intervention on physical activity and functioning after coronary artery bypass surgery among older adults with high disease burden. Heart & Lung 38:459–468 19944870 10.1016/j.hrtlng.2009.01.010 PMC2841300

[51] Zimmerman L, Barnason S, Schulz P, Nieveen J, Miller C, Hertzog M et al (2007) The effects of a symptom management intervention on symptom evaluation, physical functioning, and physical activity for women after coronary artery bypass surgery. J Cardiovasc Nurs 22:493–500 18090191 10.1097/01.JCN.0000297379.06379.b6

[52] Zimmerman L, Barnason S, Hertzog M, Young L, Nieveen J, Schulz P et al (2011) Gender differences in recovery outcomes after an early recovery symptom management intervention. Heart Lung 40:429–439 10.1016/j.hrtlng.2010.07.018 21501872 PMC3166972

[53] Heikkinen K, Salantera S, Leppanen T, Vahlberg T, Leino-Kilpi H (2012) Ambulatory orthopaedic surgery patients' emotions when using different patient education methods. J Perioper Pract 22:226–231 22919767 10.1177/175045891202200703

[54] Heikkinen K, Salanterä S, Suomi R, Lindblom A, Leino-Kilpi H (2011) Ambulatory orthopaedic surgery patient education and cost of care. Orthopaedic Nursing 30:20–28 10.1097/NOR.0b013e318205747f 21278551

[55] Young L, Zimmerman L, Pozehl B, Barnason S, Wang H (2012) Cost-effectiveness of a symptom management intervention: improving physical activity in older women following coronary artery bypass surgery. Nurs Econ 30:94–103 22558727 PMC4636338

[56] Flodgren G, Rachas A, Farmer AJ, Inzitari M, Shepperd S (2015) Interactive telemedicine: effects on professional practice and health care outcomes. Cochrane Database Syst Rev 9:CD002098 10.1002/14651858.CD002098.pub2 26343551 PMC6473731

[57] Ekeland AG, Bowes A, Flottorp S (2010) Effectiveness of telemedicine: a systematic review of reviews. Int J Med Inform 79:736–771 10.1016/j.ijmedinf.2010.08.006 20884286

[58] de lT-D I, Lopez-Coronado M, Vaca C, Aguado JS, de CC (2015) Cost-utility and cost-effectiveness studies of telemedicine, electronic, and mobile health systems in the literature: a systematic review. Telemed J E Health 21:81–85 10.1089/tmj.2014.0053 25474190 PMC4312789

[59] Eland-de KP, van Os-Medendorp H, Vergouwe-Meijer A, Bruijnzeel-Koomen C, Ros W (2011) A systematic review of the effects of e-health on chronically ill patients. J Clin Nurs 20:2997–3010 10.1111/j.1365-2702.2011.03743.x 21707807

