# Supplementary material for: The Effect of Perioperative E-Health Interventions on the Postoperative Course: A Systematic Review of Randomised and Non-Randomised Controlled Trials
Source: PLoS One. 2016 Jul 6;11(7):e0158612. doi: 10.1371/journal.pone.0158612 (PMC4934874; doi:10.1371/journal.pone.0158612)
Supplement: S1 Form — (DOCX) [file pone.0158612.s002.docx]

| **S1. Scoring form. Risk of bias scoring form - Downs and black** | | |
| --- | --- | --- |
| 1 Is the hypothesis/aim/objective of the study clearly described? | Must be explicit | Yes/No |
| 2 Are the main outcomes to be measured clearly described in the Introduction or Methods section? | If the main outcomes are first mentioned in the Results section, the question should be answered no. | Yes/No |
| 3 Are the characteristics of the patients included in the study clearly described? | In cohort studies and trials,  inclusion and/or exclusion criteria should be given. In case-control studies, a case-definition and the source for controls should be given. | Yes/no |
| 4 Are the interventions of interest clearly described? | Treatments and placebo (where relevant) that are to be compared should be clearly described. | Yes/No |
| 5 Are the distributions of principal confounders in each group of subjects to be compared clearly described? | A list of principal confounders is provided. | Yes/No |
| 6. Are the main findings of the study clearly described? | Simple outcome data (including denominators and  numerators) should be reported for all major findings so that the reader can check the major analyses and  conclusions. | Yes/No |
| 7 Does the study provide estimates of the random variability in the data for the main outcomes? | In non normally distributed data the inter-quartile range of results should be reported. In normally distributed data the standard error, standard deviation or confidence intervals should be reported. | Yes/No |
| 8. Have all important adverse events that may be a consequence of the intervention been reported? | This  should be answered yes if the study demonstrates that there was a comprehensive attempt to measure adverse events (A list of possible adverse events is provided). | Yes/No |
| 9 Have the characteristics of patients lost to follow-up been described? | This should be answered yes where there were no losses to follow-up or where losses to follow-up were so small that findings would be unaffected by their inclusion. This should be answered no where a study does not report the number of patients lost to follow-up. | Yes/No |
| 10 Have actual probability values been reported? | (e.g. 0.035 rather than <0.05) for the main outcomes except where the probability value is less than 0.001 | Yes/No |
| 11. Were the subjects asked to participate in the study representative of the entire population from which they  were recruited? | The study must identify the source population for patients and describe how the patients were selected. | Yes/No/UTD |
| 12 Were those subjects who were prepared to participate representative of the entire population from which  they were recruited? | The proportion of those asked who agreed should be stated. Validation that the  sample was representative would include demonstrating that the distribution of the main confounding factors was the same in the study sample and the source population. | Yes/No/UTD |
| 13. Were the staff, places, and facilities where the patients were treated, representative of the treatment the  majority of patients receive? | For the question to be answered yes the study should demonstrate that the  intervention was representative of that in use in the source population. The question  should be answered no if, for example, the intervention was undertaken in a specialist centre unrepresentative of the hospitals most of the source population would attend. (Must state type of hospital and  country for YES) |  |
| 14 Was an attempt made to blind study subjects to the intervention they have received? | For studies where the patients would have no way of knowing which intervention they received, this should be answered yes.  Retrospective, single group = No; UTD if > 1 group and blinding not explicitly stated | Yes/No/UTD |
| 15 Was an attempt made to blind those measuring the main outcomes of the intervention? | Must be explicit. For studies where the patients would have no way of knowing which intervention they received, this should be answered YES. | Yes/No/UTD |
| 16 If any of the results of the study were based on “data dredging”, was this made clear? | Any analyses that  had not been planned at the outset of the study should be clearly indicated. If no retrospective unplanned subgroup analyses were reported, then  answer yes. | Yes/No/UTD |
| 17 In trials and cohort studies, do the analyses adjust for different lengths of follow-up of patients, or in case control  studies, is the time period between the intervention and outcome the same for cases and controls? | Where follow-up was the same for all study patients the answer should yes. If different lengths of follow-up were adjusted for by, for example, survival analysis the answer should be yes. Studies where differences in follow-up are ignored should be answered no. | Yes/No/UTD |
| 18 Were the statistical tests used to assess the main outcomes appropriate? | The statistical techniques used  must be appropriate to the data.  The statistical techniques used must be  appropriate to the data. For example nonparametric methods should be used for small sample sizes. Where little statistical analysis has been undertaken but where there is no evidence of bias, the question should be answered yes. If the distribution of the data (normal or not) is not described it must be assumed that the estimates used  were appropriate and the question should be answered yes. | Yes/No/UTD |
| 19 Was compliance with the intervention/s reliable? | Where there was non compliance with the allocated treatment or where there was contamination of one group, the question should be answered no. For studies where the effect of any misclassification was likely to bias any association to the null, the  question should be answered yes. | Yes/No/UTD |
| 20 Were the main outcome measures used accurate (valid and reliable)? | For studies where the outcome measures are clearly described, the question should be answered yes. For studies which refer to other work or that demonstrates the outcome measures are accurate, the question should be answered as yes. | Yes/No/UTD |
| 21 Were the patients in different intervention groups (trials and cohort studies) or were the cases and controls  (case-control studies) recruited from the same population? | Patients for all comparison groups should be selected from the same hospital. The question should be answered UTD for cohort and case control studies where there is no information concerning the source of patients included in the study. | Yes/No/UTD |
| 22 Were study subjects in different intervention groups (trials and cohort studies) or were the cases and  controls (case-control studies) recruited over the same time? | For a study which does not specify the time  period over which patients were recruited, the question should be answered as UTD.  Patients for all comparison groups should be selected from the same hospital. The question  should be answered unable to determine for cohort and case control studies where there is no information concerning the source of patients included in the study. | Yes/No/UTD |
| 23 Were study subjects randomised to intervention groups? | Studies which state that subjects were  randomised should be answered yes except where method of randomisation would not ensure random  allocation. For example alternate allocation would score no because it is predictable. | Yes/No/UTD |
| 24 Was the randomised intervention assignment concealed from both patients and health care staff until  recruitment was complete and irrevocable? | All non-randomised studies should be answered no. If assignment was concealed from patients but not from staff, it should be answered no. | Yes/No/UTD |
| 25 Was there adequate adjustment for confounding in the analyses from which the main findings were  drawn? | This question should be answered no for trials if: the main conclusions of the study were based on analyses of treatment rather than intention to treat; the distribution of known confounders in the different treatment groups was not described; or the distribution of known confounders differed  between the treatment groups but was not taken into account in the analyses. In nonrandomised studies if the effect of the main confounders was not investigated or confounding was demonstrated but no adjustment was made in the final analyses the question should be answered as no. | Yes/No/UTD |
| 26 Were losses of patients to follow-up taken into account? | If the numbers of patients lost to follow-up are not reported, the question should be answered as unable to determine. If the proportion lost to follow-up was too small to affect the main findings, the question should be answered yes. | Yes/No/UTD |
| *27.* Did the study have sufficient power to detect a clinically important effect where the probability value for a difference being due to chance is less than 5%? | Answer yes when a power calculation was performed and there was sufficient power, no when a power calculation was performed, but the power was not reached or a subsample was drawn from another study and UTD when there was no report of a power calculation . | Yes/No/UTD |
