# Supplementary material for: The Effect of Perioperative E-Health Interventions on the Postoperative Course: A Systematic Review of Randomised and Non-Randomised Controlled Trials
Source: PLoS One. 2016 Jul 6;11(7):e0158612. doi: 10.1371/journal.pone.0158612 (PMC4934874; doi:10.1371/journal.pone.0158612)
Supplement: S1 Text — (DOCX) [file pone.0158612.s004.docx]

**S1. Text. Search strategies 2 dec 2015

Search strategy in PubMed (2^nd^ December 2015)**

| Search | Query | Results |
| --- | --- | --- |
| **#9** | #5 AND #8 | [**1068**](http://www.ncbi.nlm.nih.gov/pubmed/?cmd=HistorySearch&querykey=9) |
| **#8** | #6 OR #7 | [**6**](http://www.ncbi.nlm.nih.gov/pubmed/?cmd=HistorySearch&querykey=8)**79527** |
| **#7** | postoperati*[tiab] OR post-operati*[tiab] OR perioperati*[tiab] OR peri-operati*[tiab] OR preoperati*[tiab] OR pre-operati*[tiab] OR postoperati*[ot] OR post-operati*[ot] OR perioperati*[ot] OR peri-operati*[ot] OR preoperati*[ot] OR pre-operati*[ot] | **605762** |
| **#6** | "Perioperative Care"[Mesh:NoExp] OR "Perioperative Nursing"[Mesh] OR "Postoperative Care"[Mesh] OR "Preoperative Care"[Mesh:NoExp] OR "Perioperative Period"[Mesh:NoExp] OR "Postoperative Period"[Mesh] OR "Preoperative Period"[Mesh] | **155683** |
| **#5** | #1 OR #2 OR #3 OR #4 | [**10**](http://www.ncbi.nlm.nih.gov/pubmed/?cmd=HistorySearch&querykey=5)**3975** |
| **#4** | internet*[tiab] OR webbased*[tiab] OR web-based*[tiab] OR webportal*[tiab] OR web-portal*[tiab] OR world wide web*[tiab] OR internet*[ot] OR webbased*[ot] OR web-based*[ot] OR webportal*[ot] OR web-portal*[ot] OR world wide web*[ot] | [**51852**](http://www.ncbi.nlm.nih.gov/pubmed/?cmd=HistorySearch&querykey=4) |
| **#3** | "Internet"[Mesh] | [**5**](http://www.ncbi.nlm.nih.gov/pubmed/?cmd=HistorySearch&querykey=3)**5750** |
| **#2** | econsult*[tiab] OR e-consult*[tiab] OR ediagnos*[tiab] OR e-diagnos*[tiab] OR eHealth*[tiab] OR e-health*[tiab] OR mhealth*[tiab] OR m-health*[tiab] OR mobile health*[tiab] OR remote consult*[tiab] OR Teleconsult*[tiab] OR Tele-consult*[tiab] OR telediagnos*[tiab] OR tele-diagnos*[tiab] OR telehealth*[tiab] OR tele-health*[tiab] OR telemedic*[tiab] OR tele-medic*[tiab] OR telemonitor*[tiab] OR tele-monitor*[tiab] OR teleconsult*[tiab] OR tele-consult*[tiab] OR econsult*[ot] OR e-consult*[ot] OR ediagnos*[ot] OR e-diagnos*[ot] OR eHealth*[ot] OR e-health*[ot] OR mhealth*[ot] OR m-health*[ot] OR mobile health*[ot] OR remote consult*[ot] OR Teleconsult*[ot] OR Tele-consult*[ot] OR telediagnos*[ot] OR tele-diagnos*[ot] OR telehealth*[ot] OR tele-health*[ot] OR telemedic*[ot] OR tele-medic*[ot] OR telemonitor*[ot] OR tele-monitor*[ot] OR teleconsult*[ot] OR tele-consult*[ot]] | **14712** |
| **#1** | "Telemedicine"[Mesh] | **17462** |

**Search strategy in CINAHL (2^nd^ December 2015)**

| Search | Query | Results |
| --- | --- | --- |
| **S9** | S5 AND S8 | **711** |
| **S8** | S6 OR S7 | **71010** |
| **S7** | TI ( postoperati* OR post-operati* OR perioperati* OR peri-operati* OR preoperati* OR pre-operati* ) OR AB ( postoperati* OR post-operati* OR perioperati* OR peri-operati* OR preoperati* OR pre-operati* ) | **49433** |
| **S6** | (MH "Perioperative Care") OR (MH "Postoperative Care") OR (MH "Preoperative Care") OR (MH "Perioperative Nursing") OR (MH "Postoperative Period") OR (MH "Preoperative Period") | **34157** |
| **S5** | S1 OR S2 OR S3 OR S4 | **101680** |
| **S4** | TI ( internet* OR webbased* OR web-based* OR webportal* OR web-portal* OR “world wide web*” ) OR AB ( internet* OR webbased* OR web-based* OR webportal* OR web-portal* OR “world wide web*” ) | **19547** |
| **S3** | (MH "Internet+") | **88609** |
| **S2** | TI ( econsult* OR e-consult* OR ediagnos* OR e-diagnos* OR eHealth* OR e-health* OR mhealth* OR m-health* OR “mobile health*” OR “remote consult*” OR Teleconsult* OR Tele-consult* OR telediagnos* OR tele-diagnos* OR telehealth* OR tele-health* OR telemedic* OR tele-medic* OR telemonitor* OR tele-monitor* OR teleconsult* OR tele-consult* ) OR AB ( econsult* OR e-consult* OR ediagnos* OR e-diagnos* OR eHealth* OR e-health* OR mhealth* OR m-health* OR “mobile health*” OR “remote consult*” OR Teleconsult* OR Tele-consult* OR telediagnos* OR tele-diagnos* OR telehealth* OR tele-health* OR telemedic* OR tele-medic* OR telemonitor* OR tele-monitor* OR teleconsult* OR tele-consult* ) | **4305** |
| **S1** | (MH "Telemedicine+") | **4278** |

**Search strategy in Embase.com (2^nd^ December 2015)**

| Search | Query | Results |
| --- | --- | --- |
| **#9** | #5 AND #8 | **1876** |
| **#8** | #6 OR #7 | **893397** |
| **#7** | postoperati*:ab,ti OR (post NEXT/1 operati*):ab,ti OR perioperati*:ab,ti OR (peri NEXT/1 operati*):ab,ti OR preoperati*:ab,ti OR (pre NEXT/1 operati*):ab,ti | **782443** |
| **#6** | 'perioperative period'/exp OR 'perioperative nursing'/exp OR 'postoperative period'/de OR 'postoperative care'/exp OR 'preoperative period'/de OR 'preoperative care'/exp OR 'preoperative education'/exp | **288322** |
| **#5** | #1 OR #2 OR #3 OR #4 | **146044** |
| **#4** | internet*:ab,ti OR webbased*:ab,ti OR (web NEXT/1 based*):ab,ti OR webportal*:ab,ti OR (web NEXT/1 portal*):ab,ti OR ('world wide' NEXT/1 web*):ab,ti | **67767** |
| **#3** | 'internet'/exp | **81597** |
| **#2** | econsult*:ab,ti OR (e NEXT/1 consult*):ab,ti OR ediagnos*:ab,ti OR (e NEXT/1 diagnos*):ab,ti OR ehealth*:ab,ti OR (e NEXT/1 health*):ab,ti OR mhealth*:ab,ti OR (m NEXT/1 health*):ab,ti OR (mobile:ab,ti AND health*:ab,ti) OR (remote NEXT/1 consult*):ab,ti OR telediagnos*:ab,ti OR (tele NEXT/1 diagnos*):ab,ti OR telehealth*:ab,ti OR (tele NEXT/1 health*):ab,ti OR telemedic*:ab,ti OR (tele NEXT/1 medic*):ab,ti OR telemonitor*:ab,ti OR (tele NEXT/1 monitor*):ab,ti OR teleconsult*:ab,ti OR (tele NEXT/1 consult*):ab,ti | **26870** |
| **#1** | 'telemedicine'/exp | **23021** |

**Search strategy in The Cochrane Library (2^nd^ December 2015)**

| Search | Query | Results |
| --- | --- | --- |
| **#5** | #3 and #4 | **124** |
| **#4** | (postoperati* or post-operati* or perioperati* or peri-operati* or preoperati* or pre-operati*):ti,ab,kw | **78509** |
| **#3** | #1 or #2 | **7882** |
| **#2** | (internet* or webbased* or web-based* or webportal* or web-portal* or "world wide web*"):ti,ab,kw | **5612** |
| **#1** | (econsult* or e-consult* or ediagnos* or e-diagnos* or eHealth* or e-health* or mhealth* or m-health* or "mobile health*" or "remote consult*" or Teleconsult* or Tele-consult* or telediagnos* or tele-diagnos* or telehealth* or tele-health* or telemedic* or tele-medic* or telemonitor* or tele-monitor* or teleconsult* or tele-consult*):ti,ab,kw | **2749** |
